# Adverse Events Associated with Yoga: A Systematic Review of Published Case Reports and Case Series

**DOI:** 10.1371/journal.pone.0075515

**Published:** 2013-10-16

**Authors:** Holger Cramer, Carol Krucoff, Gustav Dobos

**Affiliations:** 1 Department of Internal and Integrative Medicine, Kliniken Essen-Mitte, Faculty of Medicine, University of Duisburg-Essen, Essen, Germany; 2 Duke Integrative Medicine, Duke University, Durham, North Carolina, United States of America; Casey Eye Institute, United States of America

## Abstract

While yoga is gaining increased popularity in North America and Europe, its safety has been questioned in the lay press. The aim of this systematic review was to assess published case reports and case series on adverse events associated with yoga. Medline/Pubmed, Scopus, CAMBase, IndMed and the Cases Database were screened through February 2013; and 35 case reports and 2 case series reporting a total of 76 cases were included. Ten cases had medical preconditions, mainly glaucoma and osteopenia. Pranayama, hatha yoga, and Bikram yoga were the most common yoga practices; headstand, shoulder stand, lotus position, and forceful breathing were the most common yoga postures and breathing techniques cited. Twenty-seven adverse events (35.5%) affected the musculoskeletal system; 14 (18.4%) the nervous system; and 9 (11.8%) the eyes. Fifteen cases (19.7%) reached full recovery; 9 cases (11.3%) partial recovery; 1 case (1.3%) no recovery; and 1 case (1.3%) died. As any other physical or mental practice, yoga should be practiced carefully under the guidance of a qualified instructor. Beginners should avoid extreme practices such as headstand, lotus position and forceful breathing. Individuals with medical preconditions should work with their physician and yoga teacher to appropriately adapt postures; patients with glaucoma should avoid inversions and patients with compromised bone should avoid forceful yoga practices.

## Introduction

Yoga is rooted in Indian philosophy and has been a part of traditional Indian spiritual practice for around 3000 years [1]. While the goal of yoga has been described as uniting mind, body, and spirit, it has now become a popular means to promote physical and mental well-being [1], [2]. While yoga traditionally also comprises advice for ethical lifestyle and spiritual practice [1]–[4], it is most often associated with physical postures (asanas), breathing techniques (pranayama), and meditation (dyana) in North America and Europe [2]. These more physically-oriented yoga forms are gaining increased popularity as a therapeutic practice: in 2008, about 15% of the American adult population reported practicing yoga or being at least strongly interested in it [5]. Of those who were already practicing yoga, about half started practicing explicitly to improve their health status, resulting in more than 13 million people practicing yoga for health reasons [6], [7]. It has been estimated that about 30 million people are regularly practicing yoga worldwide [8]. Yoga has also been recognized as medical therapy: about 14 million Americans (6.1% of the population) reported that yoga was recommended to them by a physician or other therapist [5].

While yoga has often been regarded as beneficial and without harm, this view has been challenged in recent years. Mainly based on anecdotal evidence, the safety of yoga has been questioned in a number of lay-press articles [9]–[11]. In particular, a recent New York Times article by William J. Broad has listed a number of alarming cases of yoga-associated injuries [11]. As these publications seem to have led to a general uncertainty among yoga practitioners and those interested in starting practice [12], it is important to systematically assess the safety of yoga. Therefore, this review aims to assess published case reports and case series on yoga-associated adverse events in order to analyze a) which adverse events were most often reported, b) which yoga forms and specific practices were most often associated with adverse events, and c) which persons (e.g. those which specific preconditions) were most often reported to be affected.

## Materials and Methods

### Eligibility Criteria

Original English or German language case reports and case series were eligible if they were published in a peer-reviewed journal and reported on yoga-associated adverse events in healthy humans or human patients. Non-case reports such as clinical trials, reviews, basic research, or commentaries were excluded. A specific practice was regarded as ‘yoga’ if a) it was explicitly labeled as yoga by the authors, b) it was labeled with the name of a specific yoga practice, and/or c) the described practice clearly resembled typical yoga practices. Adverse events were classified as yoga-associated if they appeared in temporal connection with yoga practice and/or a causal relationship was assumed by the authors of the report.

### Search Methods

An exploratory search in Pubmed was conducted on February 10, 2013 using the following search strategy: *(Yoga[MeSH Terms] OR Yoga[Title/Abstract] OR Yogic[Title/Abstract] OR Asana[Title/Abstract] OR Pranayama[Title/Abstract]) AND (Case Reports[Publication Type] OR Case[Title/Abstract] OR Cases[Title/Abstract] OR Adverse[Title/Abstract])*. Abstracts identified during this initial literature search were screened and adverse events that were associated with yoga practice in the retrieved abstracts were included in the final search strategy. In this search the following electronic databases were searched from their inception through February 15, 2013: Medline/Pubmed, Scopus, CAMBase, IndMed, and the Cases Database. The complete search strategy for each database is shown in table 1. Reference lists of identified original articles or reviews were searched manually. Additionally, the tables of contents of the International Journal of Yoga Therapy and the Journal of Yoga & Physical Therapy were reviewed.

**Table 1:** Search strategy.

PubMed	
#1	Yoga[MeSH Terms] OR Yoga[Title/Abstract] OR Yogic[Title/Abstract] OR Asana[Title/Abstract] OR Pranayama[Title/Abstract]
#2	Case Reports[Publication Type] OR Case[Title/Abstract] OR Cases[Title/Abstract] OR Adverse[Title/Abstract]
#3	Hematoma[Mesh] OR Hematoma[Title/Abstract] OR Purpura[Mesh] OR Purpura[Title/Abstract] OR Rupture[Mesh] OR Rupture[Title/Abstract] OR Myositis[Mesh] OR Myositis[Title/Abstract] OR Lymphocele[Mesh] OR Lymphocele[Title/Abstract] OR Occlusion[Title/Abstract] OR Embolism[Mesh] OR Embolism[Title/Abstract] OR Thrombosis[Mesh] OR Thrombosis[Title/Abstract] OR Stroke[Mesh] OR Stroke[Title/Abstract] OR Psychotic Disorders[Mesh] OR Psychosis[Title/Abstract] OR Psychotic[Title/Abstract] OR Pneumothorax[Mesh] OR Pneumothorax[Title/Abstract] OR Glaucoma[Mesh] OR Glaucoma[Title/Abstract] OR Neuropathy[Title/Abstract] OR Footdrop[Title/Abstract]
#4	#2 OR #3
#5	#1 AND #4
**Scopus**	
#1	TITLE-ABS-KEY(yoga) OR TITLE-ABS-KEY(yogic) OR TITLE-ABS-KEY(asana) OR TITLE-ABS-KEY(pranayama)
#2	TITLE-ABS-KEY(case) OR TITLE-ABS-KEY(cases)
#3	TITLE-ABS-KEY(hematoma) OR TITLE-ABS-KEY(purpura) OR TITLE-ABS-KEY(rupture) OR TITLE-ABS-KEY(myositis) OR TITLE-ABS-KEY(lymphocele) OR TITLE-ABS-KEY(occlusion) OR TITLE-ABS-KEY(embolism) OR TITLE-ABS-KEY(thrombosis) OR TITLE-ABS-KEY(stroke) OR TITLE-ABS-KEY(neuropathy) OR TITLE-ABS-KEY(footdrop) OR TITLE-ABS-KEY(glaucoma) OR TITLE-ABS-KEY(pneumothorax) OR TITLE-ABS-KEY(psychosis) OR TITLE-ABS-KEY(psychotic)
#4	#2 OR #3
#5	#1 AND #4
**IndMed**	
#1	(yoga OR Yogic OR asana OR pranayama) AND (Case OR hematoma OR purpura OR rupture OR myositis OR lymphocele OR Occlusion OR embolism OR thrombosis OR Stroke OR Neuropathy OR Footdrop OR Glaucoma OR Pneumothorax OR Psychosis OR Psychotic)
**CAMBase**	
#1	yoga AND (case OR hematoma OR purpura OR rupture OR myositis OR lymphocele OR occlusion OR embolism OR thrombosis OR stroke OR neuropathy OR footdrop OR Glaucoma OR pneumothorax OR psychosis OR psychotic)
**Cases Database**	
#1	yoga OR yogic OR asana OR pranayama

### Data Extraction and Management

For case reports, data were extracted on time of publication, country of origin, age and gender of the case, the specific yoga practice and yoga posture or breathing techniques, and the experience of the practitioner. Data on the reported adverse event, its treatment and clinical outcome were also extracted. For case series, the time of publication, origin, number of cases, the cases age and gender, the specific yoga practice and yoga posture or breathing techniques, the reported adverse event, its treatment and clinical outcome were collected.

## Results

### Literature Search

**Figure 1:**
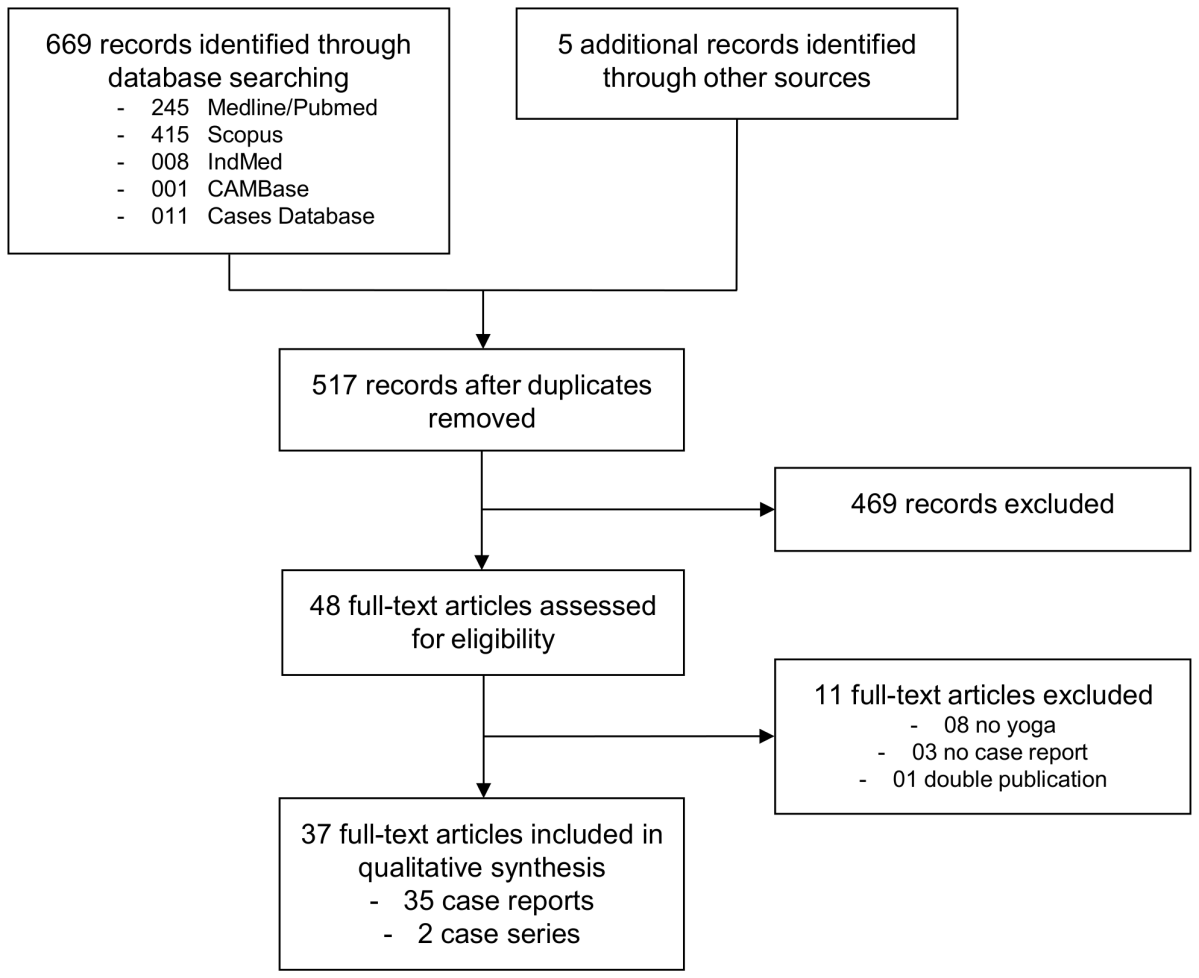
Flowchart of the literature search.

The literature search revealed a total of 517 non-duplicate records of which 469 were excluded because they did not report on yoga practices, were not case reports or case series or did not report adverse events. Out of 48 full-texts assessed for eligibility, 11 articles were excluded because they were not on yoga [13]–[20], were not case reports or case series [21]–[23], or were double publications on the same case [24]. Finally, 35 case reports [25]–[59] and 2 case series reporting on a total of 76 unique cases were included [60], [61] (Figure 1).

### Reported Cases

Characteristics of the included case reports and case series are shown in table 2 and table 3, respectively.

**Table 2:** Characteristics of the included case reports.

Reference	Case age/gender	Yogapractice	Length of practice	Yoga posture/breathing technique	Adverse event	Treatment/Clinical outcome
Bertschinger et al., 2007	46/female	Hatha yoga	1 year	Sirsasana (headstand)	Worsening of glaucomatous visual field defects	Avoiding sirasana/full recovery in several months
Bianchi et al., 2004	14/female	Unclear	Unclear	Padmasana (lotus position)	Epiphyseal fracture-separation of the distal tibia	Immobilization/full recovery in 7 weeks
Biswas et al., 2002	30/male	Unclear	Unclear	Kunjal kriya (voluntary vomiting) and Sirsasana (headstand)	Intermittent reflux symptoms	Avoiding kunjal kriya and sirsasana/almost full recovery in 6 months
Brauer et al., 2011	59/male	Vinyasa yoga	18 months	Sirsasana (headstand)	Progressive pigmentary purpura of the forehead	Topical corticosteroids/clinical outcome unclear
Chakraborty et al., 2011	Middle-aged/male	Unclear	Unclear	Extreme yoga postures	Superficial thrombophlebitis	Ibuprofen and heparinoid ointment/full recovery in 2 weeks
Choi et al., 2009	45/female	Unclear	Unclear	Unclear	Rectus sheath hematoma	Inpatient treatment with pain control, fluid therapy/full recovery in 9 days
Chusid, 1971	22/male	Hatha yoga	18 months	Vajrasa (kneeling pose)	Bilaeral peroneal neuropathy	Thiamine hydrochlorid/full recovery in 9 weeks
Cohen et al., 1995	62/female	Unclear	Unclear	Sirsasana (headstand)	Bilateral orbital varices	Unclear
Corrigan et al., 1969	16/female	Unclear	Unclear	Voluntary mouth-to-mouth yoga breathing	Pneumomediastinum	Vasopressure drugs, intubation, reanimation/fatality
Dacci et al., 2012	67/female	Unclear	Unclear	Both feet behind neck (accidentally lost balance)	Bilateral sciatic nerve neuropathy in congenital hyperelasticity of connective tissue	Steroids/parital recovery after 4 months
Fahmy & Fledelius, 1973	47/female	Unclear	6 months	Sarvangasana (shoulder stand) and Salabhasana (locust pose)	Acute glaucoma	Iridectomy/full recovery in 6 months
Fong et al., 1993	34/female	Unclear	2 months	Sirsasana (headstand)	Basilar artery occlusion	Inpatient treatment, physiotherapy/almost full recovery in 1 year
Gallardo et al., 2006	46/female	Unclear	10 years	Sirsasana (headstand)	Progressive optic neuropathyin a patient with glaucoma	Unclear
Hanus et al., 1977	25/male	Unclear	18 months	Head hyperrotation and Sarvangasana(shoulder stand)	Vertebral artery occlusion	Inpatient treatment, physiotherapy/partial recovery in 2 months
Johnson et al., 2004	29/female	Pranayama	Unclear	Kapalabhati (breath of fire)	Pneumothorax	Inpatient treatment with chest tube/full recovery in 7 days
Kashyap et al., 2006	40/male	Pranayama	Unclear	Exercise involing a vigorous Valsalva manoeuvre	Pneumomediastinum	Unclear
Khalil, 2008	42/male	Unclear	Unclear	Unclear	Lymphocele	Surgery/outcome unclear
Kim et al., 2010	30/female	Bikram yoga	5 weeks	Unclear	Rosacea	Oral minocycline and topical treatment/partial recovery
Kohanzadeh et al., 2012	38/female	Unclear	Unclear	Unclear	Myositis ossificans of the forearm	Non-steroidal anti-inflammatory drugs, surgery, hand therapy/complete recovery after 8 weeks
Lu & Pierre, 2007	33/male	Bikram yoga	Unclear	Unclear	Psychotic episode	Aripiprazole/full recovery in 1 month
Margo et al., 1992	60/male	Unclear	10 years	Sirsasana (headstand)	Bilateral conjunctival varix thromboses	Surgical excision/clinical outcome unclear
Mattio et al., 1992	38/male	Siddha yoga meditation	15 years	Padmasana(full lotus position)	Lateral femoral cutaneous neuropathy	Unclear
Meshramkar et al., 2007	38/male	Hatha yoga	12 years	Kunjal kriya (voluntary vomiting)	Dental erosion	Dental crowns, giving up kunjal kriya/full recovery
Monteiro de Barros et al., 2008	47/female	Unclear	5 years	Sirsasana (headstand)	Progressive optic neuropathyin a patient with congenital glaucoma	Unclear
Nagler, 1973	28/female	Unclear	Unclear	Setu bandha (Bridge pose)	Vertebral artery occlusion	Extensive rehabilitation program/Partial recovery in 2 years
Patel & Parker, 2008	34/male	Unclear	Unclear	Head behind foot pose	Collateral ligament rupture	Non-operative treatment/full recovery in 12 months
Reynolds er al., 2012	34/female	Bikram yoga	Unclear	Unclear	Hyponatraemia	Inpatient treatment with hypertonic saline infusion/full recovery in 5 days
Rice and Allen, 1985	29/male	Unclear	Several years	Sirsasana (headstand) and Sarvangasana (shoulder stand)	Early glaucomatous optic disk change and visual field loss	Avoiding inversions/stable ocular status
Shah and Shah, 2009	55/male	Unclear	2 years	Sirsasana (headstand)	Central retinal vein occlusion	Pan-retinal laser photocoagulation/no recovery
Sharma et al., 2007	61/female	Pranayama	Unclear	Unclear	Rectus sheath hematoma	Surgery/full recovery in 4 days
Tamarin et al., 1988	63/male	Yoga breathing	Unclear	Forceful repetetive undulating movements	Increased serum muscle enzymes in asthmatic patient	Nebuliser treatment/full recovery
Vogel et al., 1991	20/female	Siddha yoga	Unclear	Modified padmasana (lotus position)	Sciatic neuropathy	Treatment unclear/almost full recovery in 4 months
Walker et al., 2005	42/female	Unclear	Unclear	Paschimottana (seated forward bend) (fell asleep for 4 hours due to Oxycodone and amitriptilyn use)	Acute bilateral sciatic nerve compression neuropathy	Physical therapy and assistive orthotics/partial recovery in 3 months
Yeh et al., 2011	52/female	Unclear	10 years	Adho Mukha Svanasana (downward-facing dog)	Common flexor tendon tear	Surgical repair/partial recovery in 3 months
Yorston, 2001	25/female	Unclear	Unclear	Unclear	Manic episode	Haloperidol and Lorazepam/full recovery in 8 weeks

**Table 3:** Characteristics of the included case series.

Reference	Numberof cases	Cases age/gender	Yoga practice	Yoga posture/breathing technique	Adverse events	Treatment/Clinical outcomes
Le Coroller et al., 2012	38	19–67/female (n = 28), male (n = 10)	Unclear	Unclear	Fibrocartilaginous injuries (n = 8), medialmeniscus tears (n = 2), acetabular labrumtears (n = 2), lumbar disk annular tears(n = 2), transient patellar dislocation (n = 2),dissociation of the polyethylene liner fromthe acetabular cup in total hip replacement(n = 1), inguinal hernia (n = 1), great toefracture (n = 1), solitary joint effusion (n = 2),transiant headache (n = 7), unclear (n = 11)	Unclear
Sinaki, 2012	3	61–87/female (n = 3)	Unclear	Spinal flexion exercise	Vertebral compression fracture inosteopenia patients (n = 3)	Unclear

**Figure 2:**
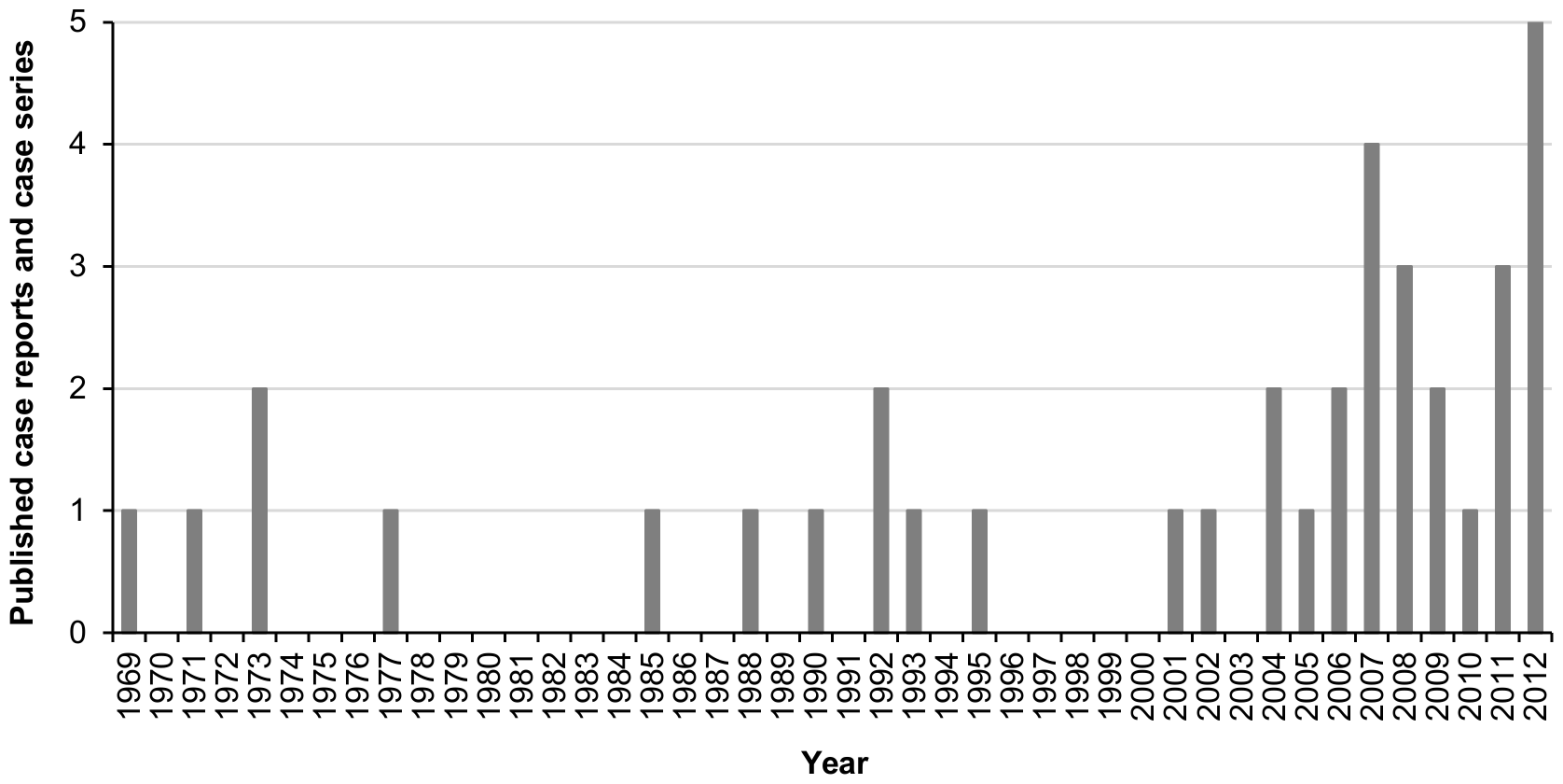
Number of published case reports and case series in a given year.

Of the included 37 reports, 19 originated from the USA [28], [31]–[33], [35], [38]–[40], [43]–[47], [49], [52], [55]–[57], [61], 1 from Canada [60], 2 from the UK [51], [59], 1 from Germany [42], 1 from Switzerland [25], 2 from Italy [26], [34], 1 from Denmark [36], 5 from India [29], [41], [48], [53], [54], and 1 each from Nepal [27], China [37], Taiwan [58], South Korea [30], and Australia [50]. The first included report was published in 1969, the number of reports published each year gradually increased until 2012 (Figure 2).

Of the 76 cases, 66 had no preconditions that were associated with the adverse events while 9 case reports described an aggravation of existing preconditions, i.e. 3 cases of glaucoma [25], [35], [38], 3 cases of osteopenia [61], and 1 case each of asthma [55], psychosis [45], and affective disorder [59]. One case had a congenital hyperelasticity of connective tissue which might have facilitated the occurence of adverse events [34]. Fifty-one cases were female, 25 male; the mean age was 44.23 years.

The yoga practice that was most often associated with reported adverse events was Pranayama or yoga breathing with 4 reported cases [40], [41], [54], [55], followed by Hatha yoga (an umbrella term for physical yoga practices) [25], [31], [48] and Bikram yoga [43], [45], [51] with 3 cases each. Siddha yoga meditation [47], [56] and Vinyasa yoga (a yoga practice that involves flowing sequences of yoga postures synchronized to the breath) [28] were practiced in 2 and 1 cases, respectively. The other case reports or case series did not report the specific yoga practice.

Regarding specific yoga postures, the headstand (Sirsasana) was practiced in 10 cases [25], [27], [28], [32], [35], [37], [38], [46], [52], [53], the shoulder stand in 3 cases [36], [39], [52], variations of the lotus position (Padmasana) in 3 cases [26], [47], [56], forceful breathing techniques in 3 cases [40], [41], [55], voluntary vomiting (Kunjal Kriya) in 2 cases [27], [48], and postures that included putting 1 or 2 feet behind the head in 2 cases [34], [50]. Kneeling posture (Vajrasana) [31], locust pose (Salabhasana) [36], bridge pose (Setu bandha) [49], seated forward bend (Paschimottasana) [57], and downward-facing dog (Adho mukha savasana) [58] were practiced in 1 case each. One case of a female teenager was reported to have practiced “voluntary mouth-to-mouth Yoga breathing exercises with a teenage boy” [33]. Another case was reported doing “extreme yoga postures” that were not further characterized [29]. In 10 cases, the yoga practice that was associated with the adverse event was practiced under supervision [26], [42], [43], [45], [51], [54], [56], [58], [59], [61], in 4 cases it was unsupervised [33], [53], [55], [61]. The remaining reports did not state whether the practice was supervised or not.

In 27 cases, adverse events affected the musculoskeletal system and included fractures [26], [60], [61], ligament tears [50], [58], [60], joint injuries [60], fibrocartilaginous injuries [60], lumbar disc annular tears [60], myositis ossificans [44], and increased muscle enzymes [55]. Nine cases reported orbital adverse events including acute glaucoma [36], [52], worsening of chronic glaucoma [25], [35], [38], and orbital varices or vein occlusion [32], [46], [49], [53]. Peripheral neuropathy was reported by 4 cases [34], [47], [56], [57], stroke by 3 cases [37], [39], [49], and transient headache by 7 cases [60]. Three cases presented with pneumothorax [40], [41] or pneumomediastinum [33]. Two cases presented with rectum sheath hematoma [30], [54]. Ten further adverse events were reported just once [27]–[29], [42], [43], [45], [48], [51], [59], [60] (see tables 2, 3); the remaining 11 adverse events were unclear [60].

Fifteen cases reached full recovery without [25] or after adequate treatment [29]–[31], [36], [40], [44], [45], [48], [50], [51], [54], [55], [57], [59] and 9 cases reached partial recovery [27], [34], [37], [39], [43], [49], [52], [56], [58]. One case did not reach any recovery [53] and 1 case died [33]. In the remaining cases, clinical outcomes were not reported [28], [32], [35], [42], [46], [47], [60], [61].

## Discussion

This systematic review included 76 unique cases of yoga-associated adverse events. Most adverse events affected the musculoskeletal, nervous, or visual system. More than half of the cases for which clinical outcomes were reported reached full recovery, 1 case did not recover at all, and 1 case died. Headstand was by far the most often cited yoga posture; and Pranayama and Bikram yoga were the yoga practices that were most often associated with adverse events.

Incidence rates of adverse events associated with yoga are best estimated from large prospective surveys of practitioners. However, these data are rare. In a small survey in 110 Finnish Ashtanga Vinyasa Yoga practitioners, 62% of respondents reported at least one yoga-related musculoskeletal injury, mainly sprains and strains [62]. About half of those reported full recovery, the other half partial recovery. Ashtanga Vinyasa Yoga is a physically demanding yoga style that uses standardized sequences of physical yoga postures with synchronized breathing [62]. More recently, in a large national survey, 78.7% of about 2500 Australian yoga practitioners indicated that they had never been injured during yoga [63]. The remaining practitioners mainly reported minor injuries. 4.6% of respondents had been injured in the past 12 months; 3.4% reported injuries that occurred under supervision. In accordance with the present systematic review, the postures that were most commonly associated with injuries were headstand, shoulder stand and variations of the lotus pose [63]. A survey in more than 1300 mainly North American yoga teachers and therapists found that respondents considered injuries of the spine, shoulders, or joints the most common; many respondents regarded yoga as generally safe and associated adverse events with excessive effort, inadequate teacher training, and unknown medical preconditions [64]. Systematic reviews on clinical trials on yoga interventions generally found insufficient reporting of safety data [65]–[68]. However, if adverse events were reported, they could mostly be classified as non-serious [65]–[67].

Out of 76 cases in the present review, 1 fatality was reported [33]. However, the practice described was “voluntary mouth-to-mouth Yoga breathing exercises”, which can hardly be characterized as a typical yoga practice. This practice is not described in any standard handbook of yoga practices [1], [69]. Moreover, postmortem toxicological studies revealed significant levels of long-acting barbiturates that can be argued to be at least partially responsible for her death. Another case report reported a neuropathy being caused by falling asleep in a seated forward bend due to opioids and tricyclic antidepressants [57]. As yoga requires awareness and concentration [70], [71], it is recommended that practitioners abstain from using alcohol or recreational drugs during practice in order to avoid adverse events.

Several of the reported adverse events occurred in yoga teachers [27], [32], [45], who can be assumed to practice more intensely and more often than non-teachers. The yoga postures that were most often associated with adverse events were headstand, shoulder stand, postures that required putting 1 or both feet behind the head, and variations of the lotus position. All these postures can be considered advanced postures that should normally not be practiced by beginners or individuals with medical preconditions [1]. So-called inversions like headstand and shoulder stand are often regarded as a special category of yoga postures that should be practiced only by experienced practitioners, with extreme care. [1], [72]. Two of the 3 cases who had practiced shoulder stand [36], [52] and 8 of the 10 cases that had practiced head stand [25], [32], [35], [37], [38], [46], [52], [53] reported orbital adverse events, mainly glaucomatous symptoms. It has been reported that headstand induces a twofold increase in intraocular pressure [73]. However, intraocular pressure returned to baseline values immediately after headstand and no association of regular yoga practice with chronically increased intraocular pressure was found [73]. Therefore, beginners should be exceedingly cautious with inversions, which may be contraindicated for individuals with a history or positive family history of glaucoma.

Voluntary vomiting is a common Kriya or cleansing technique in traditional yoga [69]. It is however very rarely practiced in North America or Europe [2]. As a case of intermittent reflux symptoms [27] and another one of dental erosion [48] – both of which originated from India – can be assumed to be directly related to regular vomiting, and the postulated cleansing properties of the practice are not in accordance with biomedical science, this practice should be discouraged in general.

Further, 4 adverse events were associated with yoga breathing, or pranayama. While gentle forms of yoga breathing, such as the relaxed abdominal breath, may be appropriate for beginners, extreme forms that involve holding or forcing the breath are considered an advanced yoga practice that should not be done by those new to yoga. [1], [22], [74]. None of the respective case reports stated the length of practice of the affected individual [40], [41], [54], [55]. Yoga practitioners should be advised to be careful when practicing pranayama and perhaps not start practicing forceful techniques such as Kapalabathi, i.e. a practice that resembles hyperventilation, before they have gained a considerable body control and have mastered easier breathing techniques [1], [74]. People with medical conditions should consult their physician regarding the appropriateness of extreme breathing techniques.

Bikram yoga is a modern yoga style that includes traditional Hatha yoga practices in a room heated to 105°F with a humidity of 40% [75]. Bikram yoga is a very intense physical yoga practice that includes forceful exercise and competition [75]. At least 1 of the 3 Bikram yoga-associated adverse events, a hyponatriaemia due to excessive fluid replacement after intensive sweating [51], can be directly related to the specific conditions in Bikram yoga and cannot be transferred to other yoga styles. The extreme heat and intensity of the Bikram yoga practice may make this style of yoga inappropriate for older adults and people with medical conditions.

The majority of cases were female and the number of reports published each year gradually increased from 1969 to 2012. These findings reflect general characteristics of yoga practitioners. About 75% of all yoga users are female [76], [77] and yoga is gaining increased popularity over time: in 1994, about 5 million American Adults practiced yoga [78], by 2002, more than 10 million [7], and by 2007, more than 13 million [6]. Most cases included in this review originated from the USA. While there are no reliable data on prevalence of yoga use outside the USA, this might reflect a presumable higher prevalence of yoga use in the USA compared to most other countries worldwide [79].

There are several limitations in this review. Only case reports and case series that were published in peer-reviewed journals were included to ensure a certain quality of assessment and reporting. However, cases that were published in grey literature might have enhanced the findings of the review. Moreover, the quality of reporting in the included case reports and case series generally was low. Only few reports described the specific yoga form practiced or the practice experience of the case. Even more critically, for about 2 thirds of reported cases, no information on clinical outcomes was provided. This makes it hard to estimate the number of non-recovered or only partially recovered cases; information that is crucial for assessing the safety of yoga. Case reports and case series are anecdotal by nature. Therefore, this systematic review is unable to estimate the total number or frequency of adverse events associated with yoga.

## Conclusions

As any other physical or mental practice, yoga is not without risk. However, given the large number of practitioners worldwide [6]–[8], only relatively few serious adverse events have been reported in healthy individuals. Therefore, there is no need to discourage yoga practice for healthy people. It has however been stressed that yoga should not be practiced as a competition and that yoga teachers and practitioners should never push themselves (or their students) to their limits [9]. Beginners should avoid advanced postures such as headstand or lotus position and advanced breathing techniques such as Kapalabathi. Practices like voluntary vomiting should perhaps be avoided completely.

As yoga has been shown to be beneficial for a variety of conditions [65], [66], [68], it can also be recommended to patients with physical or mental ailments, as long as it is appropriately adapted to their needs and abilities and performed under the guidance of an experienced and medically trained yoga teacher. Especially, patients with glaucoma should avoid inversions and patients with compromised bone and other musculoskeletal disorders should avoid forceful or competitive yoga forms. Yoga should not be practiced while under the influence of psychoactive drugs.

## Supporting Information

Checklist S1 **PRISMA Checklist.**
(DOC)
